# Supplementary figures and images for: MCAK recognizes the nucleotide-dependent feature at growing microtubule ends
Source: eLife. 2025 Nov 19;12:RP92958. doi: 10.7554/eLife.92958 (PMC12629592; doi:10.7554/eLife.92958)

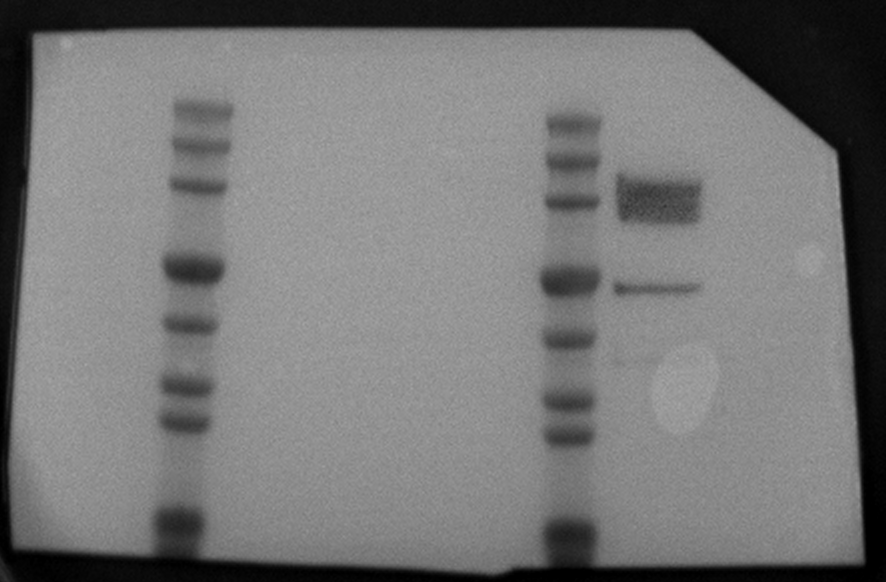

Supplement: Figure 1—figure supplement 1—source data 2. [file elife-92958-fig1-figsupp1-data2.zip › Figure1-figure supplement 1-source data2/Fig-s1B.tif]

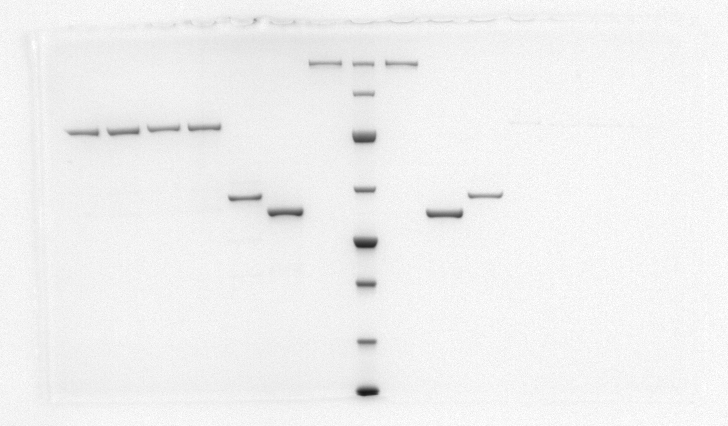

Supplement: Figure 1—figure supplement 1—source data 2. [file elife-92958-fig1-figsupp1-data2.zip › Figure1-figure supplement 1-source data2/Fig-s1J.tif]
