# Supplementary material for: MCAK recognizes the nucleotide-dependent feature at growing microtubule ends
Source: eLife. 2025 Nov 19;12:RP92958. doi: 10.7554/eLife.92958 (PMC12629592; doi:10.7554/eLife.92958)
Supplement: Figure 1—figure supplement 1—source data 3. [file elife-92958-fig1-figsupp1-data3.zip › Figure 1-figure supplement 1-source data3/Figure 1-figure supplement 1-source data3.pdf]

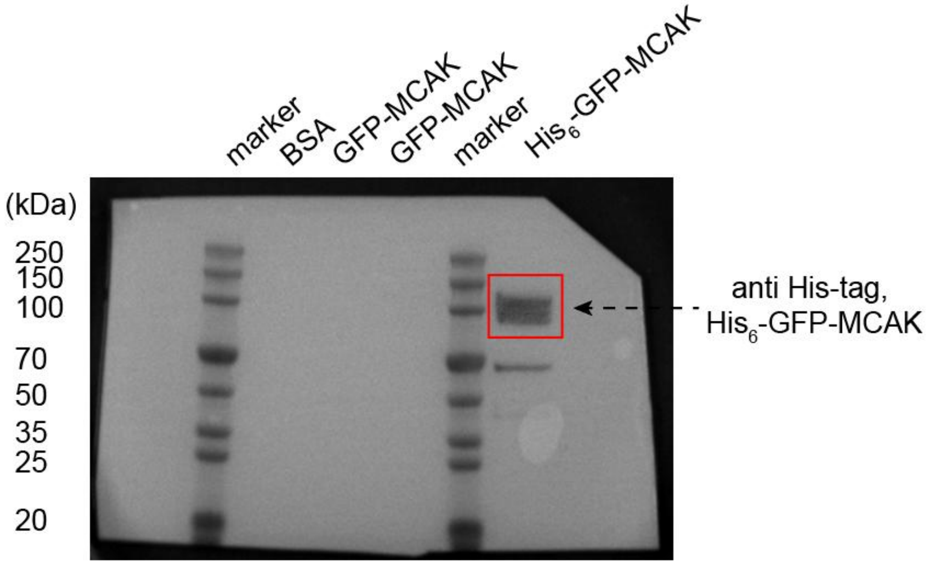


**Figure 1-figure supplement 1B.**
Detection of the His-tag by western blotting

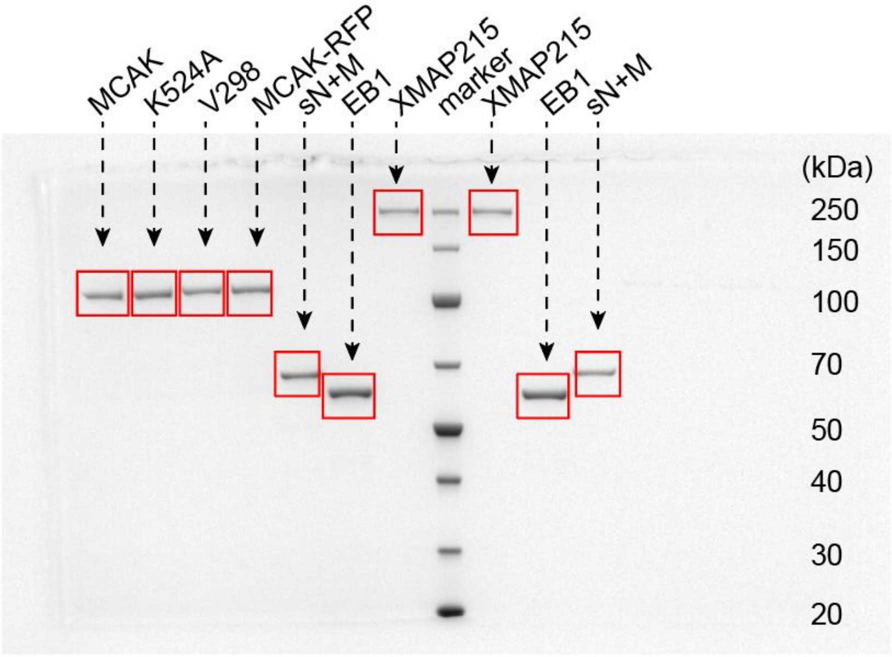


**Figure 1-figure supplement 1J.**
SDS-PAGE analysis of the proteins used in this study
